# Assessment of pediatric nurses' knowledge of budesonide–formoterol inhaler technique: a cross-sectional study in tertiary hospitals in China

**DOI:** 10.3389/fped.2026.1855978

**Published:** 2026-06-18

**Authors:** Chengjiu Tan, Qinyuan Pan, Yan Zhou, Dan Zhu, Feina Mo, Liucheng Huang, Jialing Xiang

**Affiliations:** The First Affiliated Hospital of Guangxi Medical University/Guangxi Clinical Research Center for Pediatric Diseases (Focuses on Complex and Critical Cases), Nanning, China

**Keywords:** asthma, budesonide–formoterol inhaler, inhalation device, inhalation therapy, knowledge assessment, pediatric nursing

## Abstract

**Background:**

Proper inhalation technique is essential for effective asthma management in children. Nurses play a critical role in educating patients; however, previous studies have shown variability in healthcare professionals' knowledge and inhalation device instruction skills, which may affect the quality of asthma education provided to patients.

**Objective:**

To assess pediatric nurses' knowledge regarding the use of the budesonide–formoterol inhaler and identify factors associated with higher knowledge scores.

**Methods:**

A cross-sectional survey was conducted among 57 pediatric nurses from tertiary hospitals in Nanning, Guangxi, China. A structured questionnaire based on expert consensus guidelines was used to assess theoretical knowledge regarding inhalation device use. Data were analyzed using descriptive statistics, chi-square tests, and one-way ANOVA.

**Results:**

The mean knowledge score was 85.07 ± 17.94, with 89.47% of participants achieving a score of ≥60, which was used as an institutional reference threshold for higher knowledge performance. Nurses working in specialized respiratory wards, those exposed to a higher proportion of asthma patients, and those receiving more frequent training demonstrated significantly higher scores (*P* < 0.05).

**Conclusion:**

Pediatric nurses demonstrated generally adequate knowledge of inhalation therapy; however, gaps remain. Targeted training and increased clinical exposure may improve knowledge and potential quality of patient education. The findings should be interpreted cautiously because the study was limited by convenience sampling, a small regional sample, and the lack of direct assessment of practical skills.

## 1 Introduction

Asthma is the most common chronic airway disease in childhood (1), caused by various factors such as allergen exposure, pathogen infection, or trauma, leading to acute or subacute airway inflammation that may persist or recur if not properly managed (2). Childhood asthma not only affects growth and development, but can also influence personality, psychological well-being, and behavior, and increases the risk of developing chronic obstructive pulmonary disease in adulthood, thereby adding a significant burden on families and society (3, 4). Therefore, controlling the recurrence of childhood asthma and improving quality of life remain central goals of treatment and care.

In clinical practice, pharmacological therapy is a key strategy for controlling asthma attacks, with inhalation therapy being the preferred route due to its targeted delivery to the respiratory tract (5). Budesonide–formoterol inhaler, a combination of a beta-agonist and glucocorticoid, acts on both airway inflammatory and structural cells, providing comprehensive asthma control. Its combined use improves lung function in children, enhances quality of life, and reduces adverse reactions (6, 7). It is commonly used in children over six years old for asthma management (5).

As frontline healthcare providers in inhalation therapy, nurses' ability to correctly and consistently instruct patients in proper inhalation technique directly affects treatment efficacy and disease control (8). Previous studies have reported deficiencies and inconsistencies in inhalation device knowledge and technique instruction among healthcare professionals, including nurses and physicians, particularly regarding correct operational steps and patient education practices (9, 10). Although several studies have evaluated inhalation technique knowledge among healthcare professionals, evidence specifically focusing on pediatric nurses and budesonide–formoterol inhaler use in China remains limited. Pediatric nurses are often responsible for repeated patient education and reinforcement of inhalation techniques during hospitalization; therefore, understanding their level of theoretical knowledge is important for identifying educational gaps and improving standardized asthma care. This study focused specifically on theoretical knowledge rather than attitudes or clinical practice because accurate understanding of inhalation device procedures represents the foundation for subsequent patient education and practical skill performance. Assessing knowledge gaps may help inform the development of targeted educational interventions for pediatric nurses. This study aimed to assess pediatric nurses' theoretical knowledge regarding budesonide–formoterol inhaler use and to explore factors associated with higher knowledge scores.

## 2 Data and methods

### 2.1 Study participants

A convenience sampling method was used to recruit 57 pediatric nurses from nine tertiary hospitals in Nanning, Guangxi, China. The inclusion criteria were that participants provided informed consent and voluntarily participated in the study, had at least three years of clinical experience in pediatrics, and had received standardized training in the use of the budesonide–formoterol inhalation device. Participants were excluded if they failed to complete the evaluation within the specified time of 20 min. Data collection was conducted between January 2025 and March 2025. A formal sample size calculation was not performed because this study was designed as an exploratory cross-sectional survey within participating hospitals.

### 2.2 Research instruments

#### 2.2.1 General information survey form

A structured questionnaire was designed to collect demographic and professional characteristics, including gender, age, education level, professional title, frequency of inhalation device training, and proportion of pediatric asthma patients in the ward. The full questionnaire, including all multiple-choice options, has been added as Supplementary File S1.

#### 2.2.2 Knowledge evaluation scale for inhalation therapy

The knowledge assessment tool was developed based on the 2020 Expert Consensus on the Use and Quality Control of Commonly Used Inhalation Devices for Children with Asthma (5). The scale consisted of 10 multiple-choice questions developed from the expert consensus recommendations. Each question contained four response options with one correct answer. Correct responses were awarded 10 points and incorrect responses received 0 points, yielding a maximum total score of 100. Question options were derived from standard answers in the evaluation scale and supplemented with alternative options identified through consultation with pediatric asthma specialists from nine tertiary hospitals. The questionnaire covered all key steps from device preparation to post-inhalation care, ensuring comprehensive assessment of theoretical knowledge related to inhalation therapy.

#### 2.2.3 Validity and reliability of the questionnaire

The questionnaire content was developed based on the aforementioned expert consensus and was reviewed by five senior pediatric respiratory nursing specialists for clarity, relevance, and comprehensiveness prior to distribution. Minor wording revisions were made based on expert feedback. A pilot assessment involving 10 pediatric nurses was conducted to evaluate readability and feasibility before the formal survey. Because the questionnaire was designed primarily as a criterion-referenced knowledge assessment tool derived from standardized clinical recommendations, formal psychometric validation and construct validity testing were not comprehensively performed. Internal consistency statistics such as Cronbach's alpha were not considered essential for the primary objectives of this study. Nevertheless, future studies may consider additional reliability analyses to further evaluate the measurement properties of the instrument.

### 2.3 Data collection procedure

Data were collected anonymously using the Questionnaire Star online survey platform. The questionnaire link and QR code were distributed to eligible participants through departmental nursing coordinators and professional communication groups within participating hospitals. A total of 60 questionnaires were distributed, and 57 valid responses were obtained, resulting in a response rate of 95%. Three questionnaires were excluded due to incomplete responses. Participants were allowed approximately two weeks to complete the questionnaire following distribution.

### 2.4 Statistical analysis

Data were analyzed using SPSS version 24.0. Categorical variables were summarized as frequencies and percentages, while continuous variables were presented as mean ± standard deviation (x¯ ± s). Assumptions of normality and homogeneity of variance were assessed prior to ANOVA analysis. Because of the exploratory nature and relatively small sample size of the study, multivariable regression analysis was not performed. A *p*-value of less than 0.05 was considered statistically significant.

## 3 Results

### 3.1 Knowledge scores regarding budesonide–formoterol inhaler use

The total knowledge score of the 57 pediatric nurses on the use of the budesonide–formoterol inhalation device was 85.07 ± 17.94 points. Fifty-one nurses (89.47%) achieved a total score of 60 points or higher. The threshold of ≥60 was used as an institutional reference standard for higher knowledge performance because it represents successful completion of at least 60% of the assessed knowledge items. However, this cut-off has not been externally validated and should be interpreted cautiously. Among the individual items, items 4 and 10 had the highest average scores, whereas items 3, 5, and 6 had the lowest scores, as shown in Table 1.

**Table 1:** Item-wise theoretical knowledge scores regarding budesonide–formoterol inhaler Use.

Item	Question	Score (x¯ ± s)	Rank
1	Do you need to check the inhalation technique at each visit?	8.59 ± 3.50	3
2	At what age would you choose to use the protective device?	8.77 ± 3.31	2
3	How do you know in advance that your child can use the device correctly?	8.07 ± 3.98	4
4	Is initialization necessary for the first inhalation treatment?	9.12 ± 2.85	1
5	How to judge that the drug has been inhaled into the airway?	7.54 ± 4.34	6
6	If inhalation strength is insufficient, can it be repeated?	7.71 ± 4.23	5
7	How do you know that the drug in the device is running out?	8.59 ± 3.50	3
8	Does the device need to be cleaned?	8.77 ± 3.31	2
9	In an acute asthma attack, can symptoms be alleviated by adding more drugs?	8.77 ± 3.31	2
10	Do you think gargling is important after inhaled corticosteroids?	9.12 ± 2.85	1

### 3.2 Comparison of scores by nurse characteristics

Nurses working in pediatric respiratory specialty wards scored higher than those in pediatric comprehensive wards. Nurses in wards where more than 5% of admitted patients had asthma scored higher than those in wards with fewer asthmatic patients. The 5% threshold was selected pragmatically to distinguish wards with relatively higher vs. lower routine exposure to pediatric asthma cases within participating hospitals rather than based on a predefined clinical standard. Nurses who participated more frequently in education and training on inhalation therapy also scored significantly higher than those with less frequent participation (*P* < 0.05). These differences were statistically significant, as summarized in Table 2. Some subgroup categories contained relatively small sample sizes, particularly nurses aged >40 years, and these findings should therefore be interpreted cautiously. Because only univariate analyses were performed, these statistically significant associations should not be interpreted as independent predictors of knowledge scores. Potential interrelationships among ward type, asthma patient exposure, and education frequency could not be assessed in the present study.

**Table 2:** Comparison of knowledge scores according to nurse characteristics .

Characteristic	Category	*n* (%)	Total Score	F	*P*
Age (years)	<25	6 (10.5%)	86.67 ± 10.33	0.100	0.905
	25–40	42 (86%)	85.10 ± 19.05		
	>40	2 (3.5%)	80.00 ± 0.00		
Sex	Male	5 (8.8%)	88.00 ± 8.37	4.642	0.708
	Female	52 (91.2%)	84.81 ± 18.63		
Education	Polytechnic school	7 (12.3%)	74.29 ± 17.18	1.787	0.177
	Junior college	26 (45.6%)	88.46 ± 14.61		
	Bachelor or above	24 (42.1%)	84.58 ± 20.64		
Work experience	<5 years	17 (29.8%)	83.53 ± 17.30	0.813	0.449
	5–10 years	15 (26.3%)	81.33 ± 20.99		
	>10 years	25 (43.9%)	88.40 ± 16.50		
Type of ward	Comprehensive	26 (45.6%)	76.54 ± 19.17	7.473	0.001*
	Specialist	31 (54.4%)	92.26 ± 13.34		
Proportion of asthmatic patients	<5%	29 (50.9%)	77.59 ± 18.64	5.569	0.001*
	≥5%	28 (49.1%)	92.86 ± 13.57		
Frequency of education	>once/week	10 (17.5%)	94.00 ± 8.43	5.343	0.008*
	>once/month	21 (36.8%)	90.48 ± 15.32		
	>once/year	26 (45.6%)	77.31 ± 19.71		

* indicates *P* < 0.05.

The item-wise knowledge scores are shown in Supplementary Figure S1.

## 4 Discussion

### 4.1 Knowledge scores regarding budesonide–formoterol inhaler use

The results of this survey indicated that the total knowledge score of pediatric nurses on the use of the budesonide–formoterol inhalation device was 85.07 ± 17.94 points, which indicates generally high theoretical knowledge among participating nurses. Most nurses demonstrated relatively high theoretical knowledge of inhalation device procedures, although there remains room for further improvement. Item-wise analysis showed that items 4 and 10 had the highest average scores, whereas items 3, 5, and 6 ranked lowest. Conceptually, items 4 and 10 primarily reflected routine operational and post-inhalation care procedures, which are commonly emphasized during routine nursing education. In contrast, items 3, 5, and 6 involved pre-administration assessment, recognition of effective drug delivery, and decision-making regarding repeated inhalation, which may require greater clinical judgment and practical understanding.

Several factors may explain these differences. First, the first use of the inhalation device requires initialization, which is a routine operation that can be learned directly from the operating instructions, resulting in better operational standardization. Second, inhaled corticosteroids are widely used in pediatric asthma management, and post-inhalation mouth rinsing is routinely emphasized to reduce local adverse effects such as oral candidiasis and hoarseness (11). Third, pre-administration evaluation is critical, but due to the busy clinical workload, nurses may neglect this step, potentially affecting the quality and consistency of inhalation education. Similar deficiencies in inhaler-related assessment and education practices among healthcare professionals have been reported previously (9, 12). Fourth, nurses may experience difficulty accurately evaluating whether inhaled medication has effectively entered the airway, particularly when relying solely on patient cooperation and observational indicators during pediatric administration. Finally, nurses may be uncertain about adverse reactions after repeated doses. Beta agonists in combination with glucocorticoids exert synergistic effects (12), and repeated use without consideration of pharmacokinetics and elimination half-life can result in excessive dosing and adverse events (13). These observations underscore the need for standardized operating procedures and enhanced training in inhalation therapy. Similar findings have been reported internationally, where healthcare professionals demonstrated adequate performance in routine inhalation procedures but lower proficiency in more complex assessment-related tasks and patient counseling components (9, 12). Furthermore, previous studies have shown that repeated education and practical exposure are associated with improved inhalation-related knowledge and instructional competency among healthcare providers (12, 13).

### 4.2 Analysis of factors influencing knowledge scores

These associations should be interpreted cautiously because multivariable adjustment was not performed. Consequently, the observed relationships cannot be considered independent predictors, and residual confounding among workplace characteristics and educational exposure may have influenced the findings.

#### 4.2.1 Ward types

Comprehensive wards treat a wide range of conditions, but relatively few children with asthma, limiting opportunities for demonstration and practice with the protective device. In non-specialized wards, initial guidance on inhalation techniques is often provided by physicians directly to patients and families, leaving nurses with fewer opportunities for continuous guidance, monitoring, and evaluation. This limits nurses' ability to promptly identify and correct improper device usage (14, 15).

#### 4.2.2 Proportion of asthma patients and frequency of nurse education

Nurses play a critical role in educating, advising, and directly intervening in asthma management for children and their families (16–18). Knowledge and practical skills in using the budesonide–formoterol inhalation device may be strengthened through repeated health education activities (12, 19–21). This study found that nurses participating in educational activities more than once per week scored significantly higher than those participating only once a year, indicating that reduced opportunities for health education can diminish nurses' practical knowledge and knowledge scores. Moreover, wards with fewer than 5% asthmatic patients provide fewer opportunities for hands-on practice, further reducing opportunities to reinforce inhalation-related knowledge through repeated practice. Consequently, nurses in such wards may have lower knowledge scores regarding budesonide–formoterol inhaler use. In participating hospitals, frequent education activities were typically implemented through routine nursing training sessions, departmental teaching rounds, case discussions, bedside instruction, and repeated patient education encounters during daily clinical practice.

Interestingly, work experience alone was not significantly associated with higher knowledge scores. Although nurses with more than 10 years of experience demonstrated numerically higher scores, the difference was not statistically significant. This finding suggests that asthma-specific education and repeated clinical exposure may contribute more substantially to inhalation-related knowledge than general nursing seniority. Consistent with adult learning theory, repeated experiential learning opportunities may strengthen retention and maintenance of procedural knowledge over time (21).

### 4.3 Limitations

This study has several limitations. First, the study used convenience sampling and included only 57 nurses from tertiary hospitals within a single geographic region, which may limit generalizability and introduce selection bias. In addition, inclusion criteria requiring prior standardized training and at least three years of pediatric experience may have contributed to higher overall knowledge scores. Second, the questionnaire primarily assessed theoretical knowledge rather than direct observation of inhalation technique performance; therefore, the findings should not be interpreted as a measure of clinical competency or practical skill proficiency. Third, the questionnaire underwent limited validation procedures, and formal construct validity and comprehensive psychometric testing were not performed. Fourth, because of the relatively small sample size, only univariate statistical analyses were conducted without multivariable adjustment for potential confounding factors. Finally, some subgroup analyses involved small numbers of participants and should therefore be interpreted cautiously. Effect sizes and confidence intervals were not calculated, which may limit interpretation of the magnitude of observed associations.

### 4.4 Implications for clinical practice

Although participating nurses demonstrated relatively high theoretical knowledge, several specific deficiencies were identified. Future training programs should place greater emphasis on pre-administration assessment, evaluation of effective inhalation, appropriate management of insufficient inhalation strength, and recognition of situations requiring repeated dosing. Practical teaching methods, including simulation-based demonstrations, standardized operational checklists, visual teaching aids, instructional videos, and repeated supervised practice sessions, may help strengthen understanding of these more complex procedural steps.

Additional support should be provided for nurses working in non-specialized wards with limited exposure to pediatric asthma patients. Establishing standardized inhalation education protocols and regular refresher training sessions may improve consistency in patient education practices and strengthen nurses' knowledge regarding inhalation device use.

## 5 Conclusion

Pediatric nurses demonstrated relatively high theoretical knowledge regarding budesonide–formoterol inhaler use; however, gaps remain in specific operational steps, particularly pre-administration evaluation and recognition of effective inhalation. More frequent training and greater clinical exposure were associated with higher knowledge scores. Future studies incorporating direct observation of inhalation technique practices are warranted.

## Data Availability

The original contributions presented in the study are included in the article/Supplementary Material, further inquiries can be directed to the corresponding author.

## Supplementary material

Supplementary Figure S1 Item-wise mean knowledge scores of pediatric nurses regarding budesonide–formoterol inhaler use.

Supplementary File 1 Questionnaire.
