# Supplementary figures and images for: Assessment of pediatric nurses' knowledge of budesonide–formoterol inhaler technique: a cross-sectional study in tertiary hospitals in China
Source: Front Pediatr. 2026 Jun 18;14:1855978. doi: 10.3389/fped.2026.1855978 (PMC13323249; doi:10.3389/fped.2026.1855978)

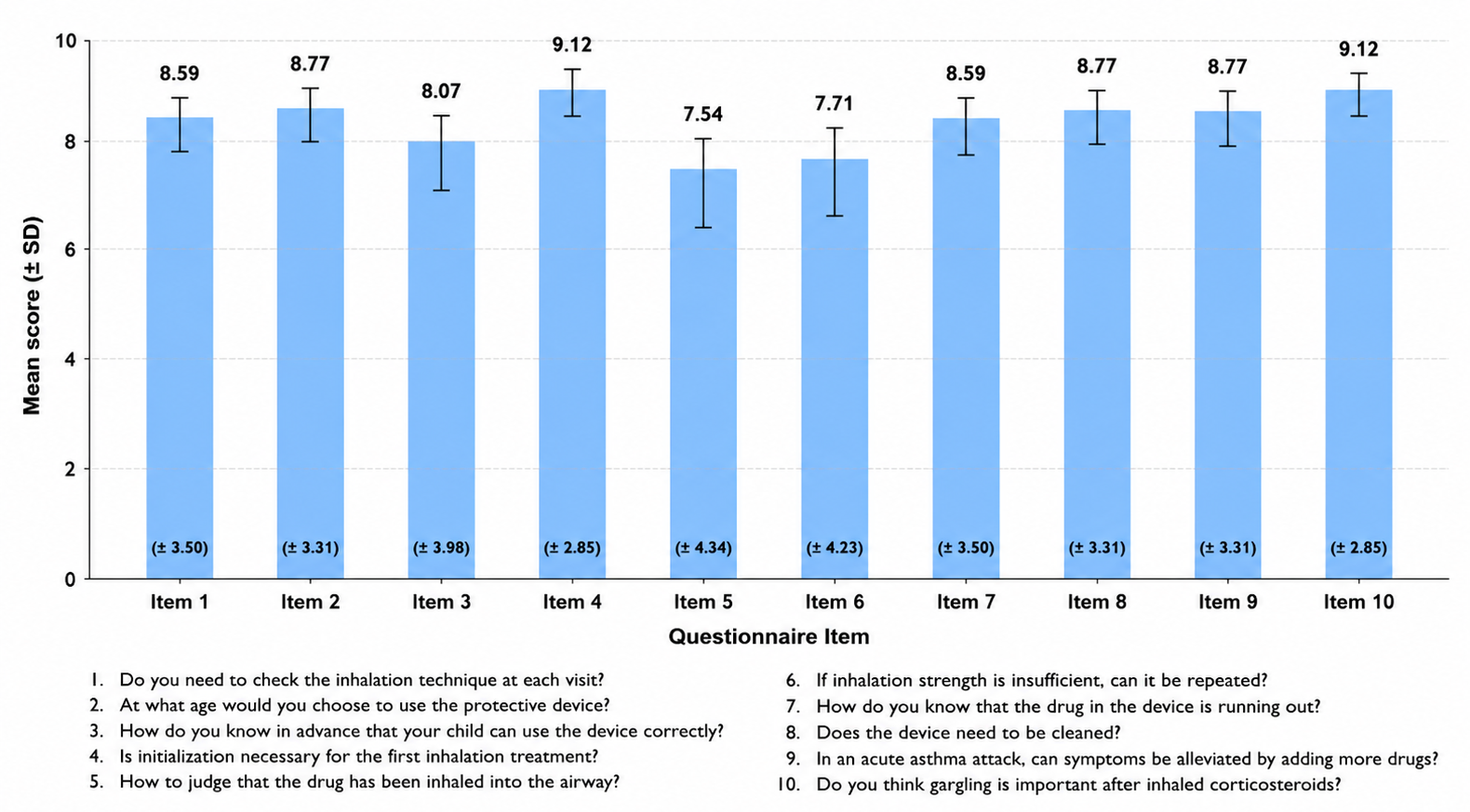

Supplement: Supplementary Figure S1 — Item-wise mean knowledge scores of pediatric nurses regarding budesonide–formoterol inhaler use. [file Image1.png]
